# Pancreatic gene expression during recovery after pancreatitis reveals unique transcriptome profiles

**DOI:** 10.1038/s41598-018-19392-0

**Published:** 2018-01-23

**Authors:** Kristy Boggs, Ting Wang, Abrahim I. Orabi, Amitava Mukherjee, John F. Eisses, Tao Sun, Li Wen, Tanveer A. Javed, Farzad Esni, Wei Chen, Sohail Z. Husain

**Affiliations:** 1 Department of Pediatrics, School of Medicine, University of Pittsburgh, Pittsburgh, PA 15224 USA; 2 Department of Surgery, School of Medicine, University of Pittsburgh, Pittsburgh, PA 15224 USA; 3 Department of Biostatistics, School of Public Health, University of Pittsburgh, Pittsburgh, PA 15224 USA

## Abstract

It is well known that pancreatic recovery after a single episode of injury such as an isolated bout of pancreatitis occurs rapidly. It is unclear, however, what changes are inflicted in such conditions to the molecular landscape of the pancreas. In the caerulein hyperstimulation model of pancreatitis, the murine pancreas has the ability to recover within one week based on histological appearance. In this study, we sought to characterize by RNA-sequencing (RNA-seq) the transcriptional profile of the recovering pancreas up to two weeks post-injury. We found that one week after injury there were 319 differentially expressed genes (DEGs) compared with baseline and that after two weeks there were 53 DEGs. Forty (12.5%) of the DEGs persisted from week one to week two, and another 13 DEGs newly emerged in the second week. Amongst the top up-regulated DEGs were several trypsinogen genes (trypsinogen 4, 5, 12, 15, and 16). To our knowledge, this is the first characterization of the transcriptome during pancreatic recovery by deep sequencing, and it reveals on a molecular basis that there is an ongoing recovery of the pancreas even after apparent histological resolution. The findings also raise the possibility of an emerging novel transcriptome upon pancreatic recovery.

## Introduction

Acute pancreatitis is a painful, life-threatening inflammatory disease that accounts for more than a quarter million hospital admissions each year in the United States and has a 20% recurrence rate^1,2^. Pancreatitis is associated with the premature activation of digestive enzymes within the pancreas and auto-digestion of the gland^3–5^. Common causes of pancreatitis include gallstones, excessive alcohol consumption, medications, and blunt trauma to the abdomen^6^. Currently, there are no targeted therapies for pancreatitis, and treatment regimens are either largely supportive or focused on reducing pancreatic inflammation. An alternative strategy to treat pancreatitis is to enhance innate recovery mechanisms of the organ. Thus, there is a need to broaden our understanding of the molecular mechanisms by which the pancreas recovers from injury.

Because obtaining clinical samples of pancreas tissue after recovery from a bout of pancreatitis is usually not feasible, much of our knowledge about pancreatic recovery after injury comes from studies using experimental animal models of pancreatic injury. Caerulein hyperstimulation is a well-characterized, non-lethal, highly reproducible rodent model of mild to moderately severe pancreatitis that mimics clinical pancreatitis^3–5,7–9^. Injury of the pancreas by caerulein hyperstimulation is marked by infiltration of inflammatory cells, edema, and destruction of more than 50% of the pancreatic parenchyma^7,8,10^. Despite the inflammatory injury and destruction of tissue, the pancreas can regenerate and recover^11,12^. Remarkably one week after injury, histologically the murine pancreas resembles that of a non-injured pancreas and is considered to have recovered^7,10,13–15^. However, it is unclear whether one week after injury, on a molecular level, the pancreas has recovered to the baseline (non-injured) state.

In this study, we examined the transcriptional changes that take place in the pancreas one and two weeks after experimental pancreatitis. To assess differentially expressed genes (DEGs), we sequenced the transcriptome of the pancreas one and two weeks after injury by RNA-seq and compared the data to the baseline pancreas. We found that by one week post-injury, compared to baseline, there were numerous DEGs, and many of these DEGs remained differentially expressed even at two weeks post-injury. There was also the emergence of unique DEGs two weeks after injury. The DEGs were associated with pancreatic secretion, digestion, the inflammatory response, cellular growth, differentiation, tissue remodeling, islet cell maintenance and function, and the translational machinery. Overall, the identification of DEGs in a histologically recovered pancreas suggests that the recovery of the pancreas takes longer than initially thought and surprisingly there is the emergence of new DEGs two weeks after injury.

## Results

### Caerulein hyperstimulation model

**Figure 1:**
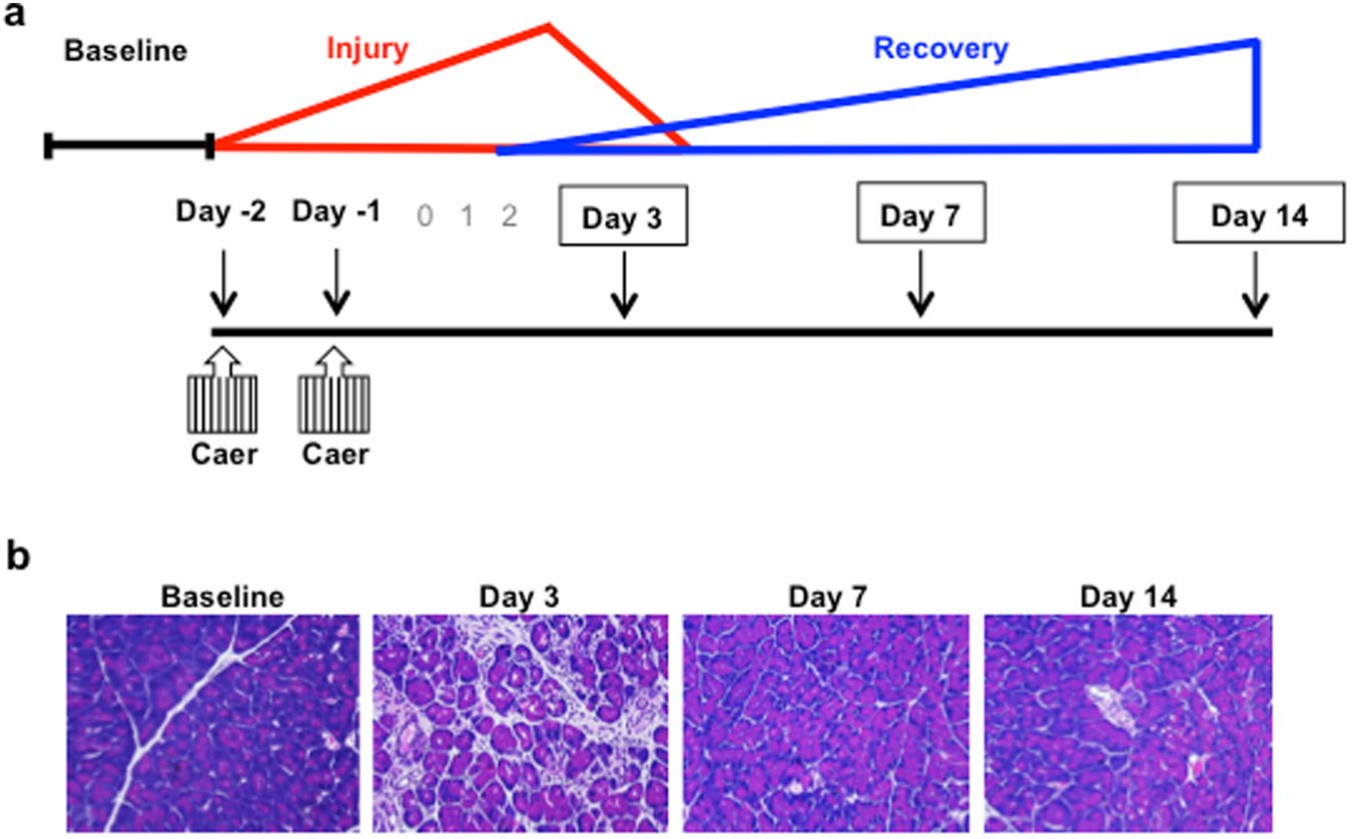
*In vivo* caerulein hyperstimulation mouse model of pancreatic injury. (**a**) Caerulein was given as 8 hourly injections for 2 days, and pancreas tissue was collected at baseline (non-injured state) and at days 3 (injury), 7 (post-injury), and 14 (post-injury). (**b**) Representative H&E staining of pancreatic sections.

A mild to moderately severe form of acute pancreatitis was induced in mice by administering 8 hourly intraperitoneal injections of caerulein for 2 consecutive days, as shown in the schema in Fig. 1a. While many reports that examine acute pancreatitis injury with caerulein use a one-day induction^16,17^, there is a precedent in the literature to study and characterize pancreatic recovery using a two-day caerulein protocol^15,18,19^. The rationale is to induce a greater degree of pancreatic parenchymal ablation so that the parenchymal recovery process is also more marked. Figure 1b illustrates pancreatic injury by hematoxylin and eosin (H&E) staining at day 3 and recovery of the pancreas at days 7 and 14 post-injury. On a histological level, by day 7 post-injury, the pancreas appears to have recovered, and it resembles the baseline state.

### Identification of DEGs in the histologically recovered pancreas

To assess at the molecular level whether the pancreas is still recovering one week after injury, we sequenced the transcriptome of the pancreas tissue by RNA-seq at baseline and at days 7 and 14 post-injury. Because the current study was intended to examine intrinsic changes in the transcriptome within pancreatic cells we intentionally avoided sequencing the transcriptome from pancreas during early recovery (i.e. less than one week post-injury) because gene expression during these early stages is confounded by infiltrating immune cells. However, at the time points we have chosen to study (i.e. one and two weeks post-injury), the immune cell infiltration has largely resolved. RNA-seq was performed on pancreas tissue rather than on cell-sorted populations. The decision not to use cell-sorted populations was to avoid eliciting DNA damage and stress responses during cell isolation. The concern was that the process of sorting could alter the transcriptome of the histologically recovered pancreas. For the sake of uniformity, only the head of the pancreas was used for RNA-seq.

**Figure 2:**
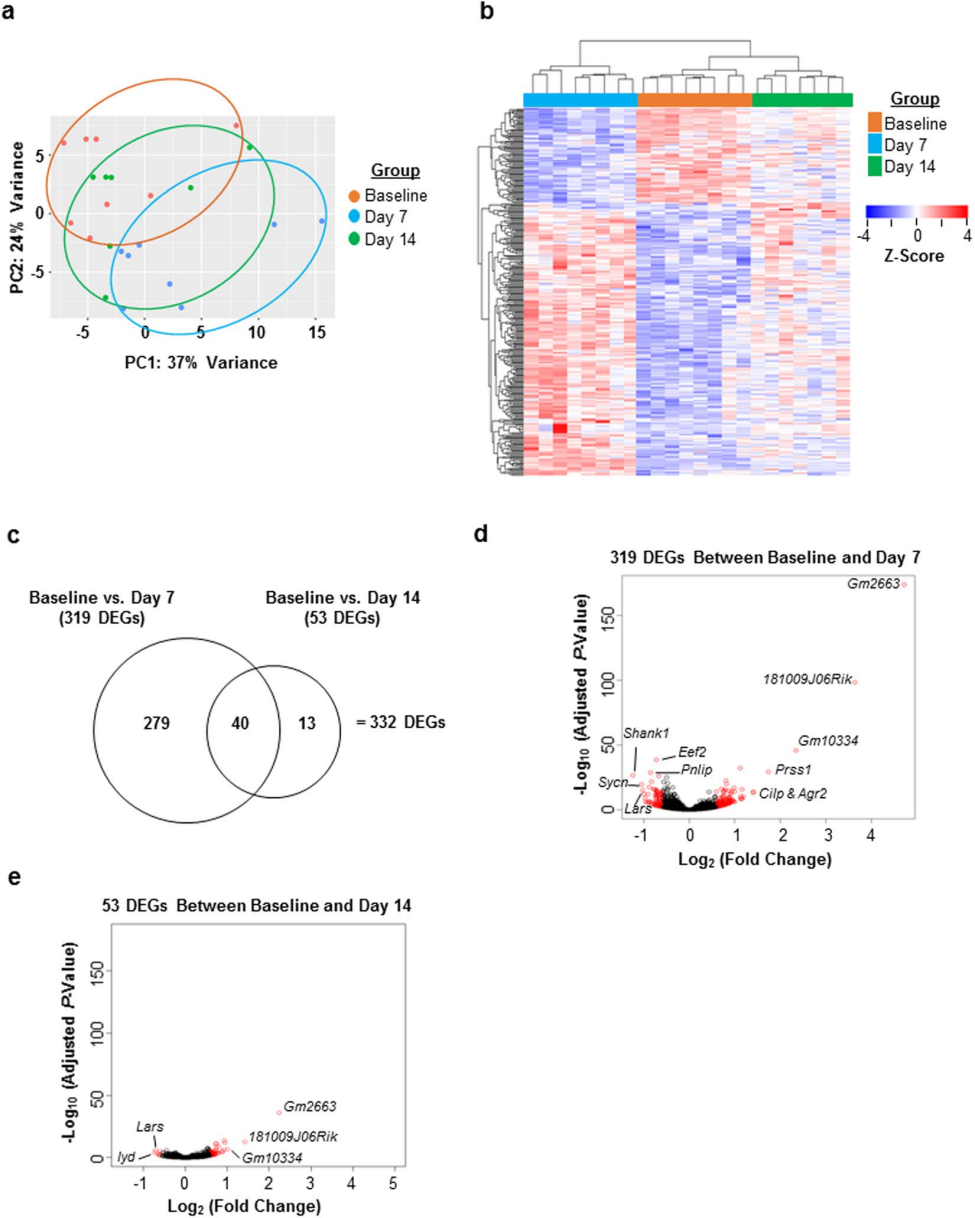
RNA-seq analysis from pancreas tissue demonstrates substantial differences in gene expression between baseline and day 7 and between baseline and day 14 post-injury. (**a**) Principal component analysis (PCA) demonstrates the variance of all genes expressed within each sample group and between each sample group. (**b**) The heat map illustrates the expression level of the DEGs between groups. Each column is a sample, and each row is a gene. The value is the z-score of normalized gene expression counts. For any given gene, the red color represents gene expression that is greater than the overall mean, and the blue color represents gene expression that is less than the overall mean. Hierarchical clustering of genes and samples are represented by the dendrograms on the left and across the top of the heat map. (**c**) Venn diagram depicting the distribution of the DEGs. (**d,e**) Volcano plots show the log_2_ (fold change) and −log_10_ (adjusted *p*-value) of all the genes in the RNA-seq dataset. The statistically significant DEGs between baseline and post-injury are represented in red. The statistical criteria for a gene to be considered differentially expressed was a fold-change ≥ 1.5 and an adjusted *p*-value ≤ 0.05.

Principal component analysis (PCA) was performed on all genes present in the RNA-seq dataset to illustrate the variance of gene expression within and between sample groups (Fig. 2a). The analysis indicated that the samples at day 14 are more like the samples at baseline compared to the samples at day 7. The data revealed a total of 332 statistically significant DEGs (fold change ≥ 1.5 and an adjusted *p*-value ≤ 0.05) between the baseline and the post-injury tissue. A heat map depicting the relative expression of each DEG in the baseline, day 7, and day 14 tissues is shown in Fig. 2b. The expression profile of the DEGs in the day 7 tissues was strikingly different from that of the baseline pancreas. Although the expression profile of the DEGs in the day 14 tissues was closer to that in the baseline pancreas compared to day 7, intriguingly the transcriptome in the day 14 pancreas still had not returned to the baseline state. The distribution of the DEGs is represented in the Venn diagram (Fig. 2c). Of the 332 DEGs identified, 279 were detected between baseline and day 7 tissues. Forty DEGs were detected in both day 7 and day 14 tissues, and 13 DEGs newly emerged in the day 14 tissue. The volcano plots show the log_2_ (fold change) versus the −log_10_ (adjusted *p*-value) for all of genes detected in the RNA-seq analysis (i.e. for both DEGs and non-DEGs; Fig. 2d,e). The statistically significant DEGs between baseline and day 7 tissues or between baseline and day 14 tissues are depicted as red circles, and the non-DEGs are depicted as black circles. The analysis indicates that even though the pancreas looks as if it has histologically recovered one week post-injury, on a molecular level there are still indicators of ongoing recovery as far out as two weeks after injury.

### Validation of the RNA-seq data

**Figure 3:**
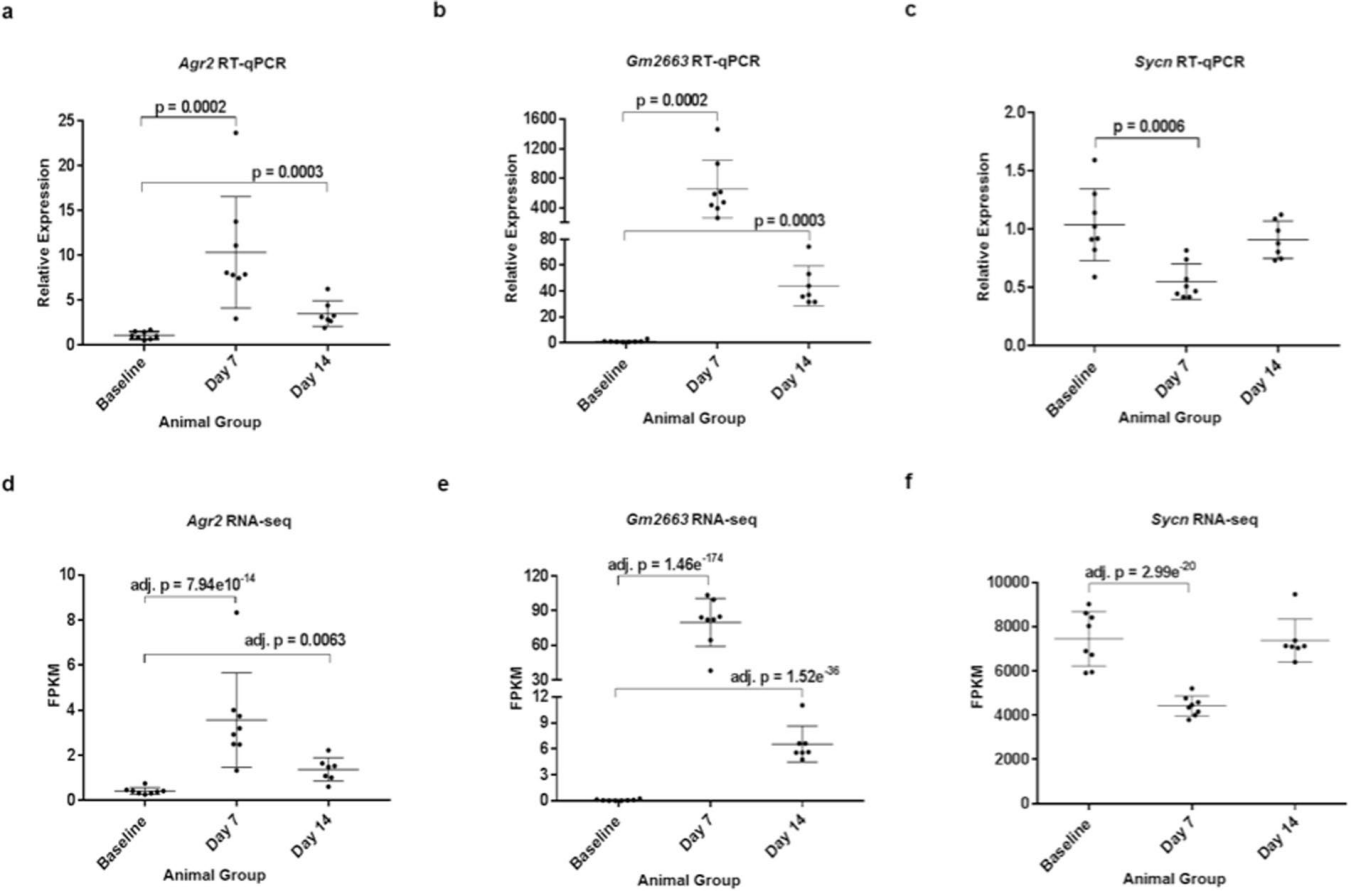
Experimental validation of DEGs from RNA-seq analysis by RT-qPCR. (**a,d**) Relative expression of *Agr2* by RT-qPCR and as FPKM values by RNA-seq. (**b,e**) Expression of *Gm2663* by RT-qPCR and RNA-seq. (**c**,**f**) Expression of *Sycn* by RT-qPCR and RNA-seq. Error bars represent ± s.d. Overall, the trend of gene expression by RT-qPCR mirrors that of the RNA-seq analysis.

To validate the data from RNA-seq, RT-qPCR was performed on DEGs with moderate and high fold change in gene expression as well as differentially repressed gene expression (Fig. 3). The patterns of relative gene expression determined by RT-qPCR closely mimicked the data from the RNA-seq analysis. Several small nucleolar RNAs (snoRNAs), which are functional non-coding RNAs that play a role in ribosomal biogenesis and thus the protein translational machinery^20^, were detected. At day 7 there were only 8 snoRNAs out of 319 DEGs, whereas at day 14 there were 12 snoRNAs out of 53 DEGs (Fig. 2c, Supplementary Tables S1 and S2). However, we were unable to validate their differential expression by RT-qPCR (Supplementary Figure S1).

### Pathway enrichment analysis

**Figure 4:**
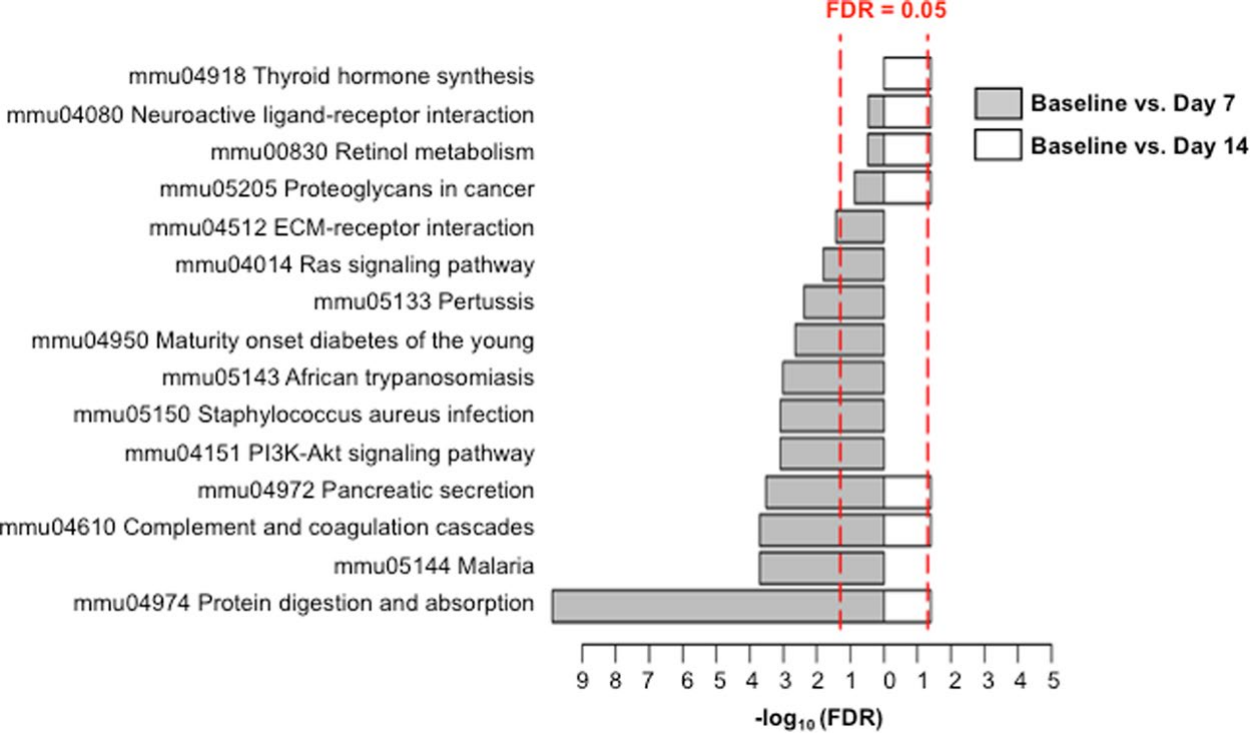
KEGG pathway enrichment analysis identifies biological pathways associated with the DEGs detected between baseline and post-injury tissue. The red dashed line represents the FDR threshold for significantly enriched pathways. On the logarithmic x-axis, an enriched pathway with an FDR ≤ 0.05 must extend to 1.3 or beyond.

KEGG pathway enrichment analysis identified molecular pathways associated with the DEGs (Fig. 4). Of the 319 DEGs detected between baseline and day 7 tissues, 104 of the genes mapped to 11 pathways. Of the 53 DEGs between baseline and day 14 tissues, 16 of the genes mapped to 7 molecular pathways. The molecular pathways associated with the enriched pathways were related to protein digestion, the inflammatory response, cell growth and differentiation, tissue remodeling, and endocrine growth. A list of the DEGs associated with each enriched molecular pathway is provided in Supplementary Table S3.

Overall, the protein digestion and absorption pathway was the most enriched pathway between baseline and day 7 tissues, and it remained significantly enriched at day 14 post-injury (Fig. 4). Many of the DEGs associated with this pathway are trypsinogens, which are zymogen precursors of the digestive enzyme trypsin. Trypsinogens are synthesized and secreted by acinar cells of the exocrine pancreas^21^. These same trypsinogens were also associated with enrichment of the pancreatic secretion pathway at both days 7 and 14 post-injury. Another pathway enriched at days 7 and 14 post-injury was the complement system, which is a mediator of the inflammatory response^22,23^.

Ras signaling, the PI3K-Akt signaling pathway, and the extracellular matrix (ECM) receptor interaction pathway were enriched at day 7 post-injury. These pathways are important modulators of cell growth, differentiation and tissue remodeling during pancreatic regeneration^24–28^. Although the Ras signaling pathway was enriched at day 7, the Ras gene itself was not found to be differentially expressed.

Several additional molecular pathways were enriched at day 14 post-injury. They included pathways for thyroid hormone synthesis, retinol metabolism, proteoglycans in cancer, and neuroactive ligand receptor interaction. While thyroid receptors (TR) are expressed in the pancreas and thyroid hormone (TH) plays a role in pancreatic cell fate and organ structure, the exact function of TR/TH in the pancreas is not well understood^29^. It has been reported that the products of thyroid metabolism, as well as retinol (Vitamin A) regulate pancreatic islet cell development, maintenance, and function^29,30^. Also, retinol and its metabolites induce quiescence in pancreatic stellate cells^31^. The DEGs found within the proteoglycans in cancer pathway are associated with pancreatic tissue repair and remodeling and the conversion of plasminogen to plasmin^32–34^.

In addition to KEGG pathway analysis, Ingenuity Pathway Analysis (IPA) was also performed to investigate the molecular functions associated with the DEGs. A list of the over-represented pathways and the DEGs associated with each pathway from the IPA are provided in Supplementary Table S4.

### The top up-regulated protein-coding DEGs

Based on the fold change, the top up-regulated protein-coding DEGs between baseline and day 7 tissues and between baseline and day 14 tissues are listed in Table 1. Among the top up-regulated DEGs at day 7 were the trypsinogens: *Prss1*, *Gm5771*, and *Prss3* (also known as trypsinogen 16, 12, and 15, respectively; Table 1). Along with trypsinogen up-regulation in the day 7 tissues, *Pi15*, a serine protease inhibitor, was also up-regulated. In the day 14 tissues, Apelin, a factor reported to dampen the inflammatory response and fibrosis during pancreatitis^35^ was significantly up-regulated. In addition, albumin was also differentially expressed in the day 14 tissue. The liver and the pancreas arise from similar endoderm progenitors, and the adult pancreas can be coaxed to express hepatocyte markers under certain conditions^36^.

**Table 1:** List of the top up-regulated protein-coding DEGs between baseline and day 7 (out of 279 DEGs, Fig. 2c) and between baseline and day 14 (out of 13 DEGs, Fig. 2c).

	Gene Symbol	Gene Name	Chromosome	Fold Change	Adjusted *P*-Value
Day 7	*Prss1*	Protease, serine 1	Chr 6	3.33	6.67E-30
	*Kcnj15*	Potassium inwardly-rectifying channel, subfamily J, member 15	Chr 16	2.24	2.57E-09
	*Gm5771*	Predicted gene 5771	Chr 6	2.22	4.98E-09
	*Prss3*	Protease, serine 3	Chr 6	2.18	2.98E-09
	*Ms4a7*	Membrane-spanning 4-domains, subfamily A, member 7	Chr 19	2.00	6.40E-07
	*Zim1*	Zinc finger, imprinted 1	Chr 7	1.99	6.76E-07
	*Col5a2*	Collagen, type V, alpha 2	Chr 1	1.98	9.98E-18
	*Ifi204*	Interferon activated gene 204	Chr 1	1.94	4.81E-08
	*Pi15*	Peptidase inhibitor 15	Chr 1	1.93	2.49E-10
	*Hpgd*	Hydroxyprostaglandin dehydrogenase 15	Chr 8	1.92	2.15E-04
Day 14	*Apln*	Apelin	Chr 10	1.62	4.55E-03
	*Akr1c14*	Aldo-keto reductase family 1, member C14	Chr 13	1.57	5.50E-03
	*Pigr*	Polymeric immunoglobulin receptor	Chr 1	1.55	2.19E-03
	*Alb*	Albumin	Chr 5	1.53	4.82E-05

The top persistently up-regulated protein-coding DEGs at day 7 that remain up-regulated at day 14 are listed in Table 2. The top persistently up-regulated DEGs were again zymogens: *Gm2663*, *1810009J06Rik*, and *Gm10334* (also known as trypsinogen 4, 5, and mesotrypsin-like, respectively). Also among the top persistent DEGs were anterior gradient 2 (*Agr2*), lumican (*Lum*), and matrix metallopeptidase 2 (*Mmp2*). These genes have been shown to regulate pancreatic tissue regeneration, ECM organization, and tissue remodeling, respectively^32,33,37^. There was persistent up-regulation of secreted frizzled-related protein 1 (*Sfrp1*), a negative modulator of Wnt signaling^38,39^. Since Wnt signaling drives acinar cell regeneration^40–42^, it is possible that SFRP1 could, by blocking Wnt, work to either keep acinar de-differentiation in check or coax re-differentiation. Also, the upregulation of *Sfrp1* during pancreatic recovery could indirectly suggest a role for zonation of acinar cell differentiation within the pancreas.

**Table 2:** List of the top up-regulated protein-coding DEGs at day 7 that remain up-regulated at day 14 (out of 40 DEGs, Fig. 2c).

Gene Symbol	Gene Name	Chromosome	Day 7		Day 14	
			Fold Change	Adjusted *P*-Value	Fold Change	Adjusted *P*-Value
*Gm2663*	Predicted gene 2663	Chr 6	26.26	1.46E-174	4.71	1.52E-36
*1810009J06Rik*	RIKEN cDNA 1810009J06Rik	Chr 6	12.40	2.68E-99	2.68	2.86E-13
*Gm10334*	Predicted gene 10334	Chr 6	5.08	1.46E-46	2.00	3.56E-07
*Cilp*	Cartilage intermediate layer protein	Chr 9	2.65	9.64E-15	1.74	3.33E-04
*Agr2*	Anterior gradient 2	Chr 12	2.65	7.94E-14	1.62	6.30E-03
*Bmper*	BMP-binding endothelial regulator	Chr 9	2.28	5.84E-11	1.84	4.03E-05
*Lum*	Lumican	Chr 10	2.23	2.85E-16	1.65	2.20E-05
*Sfrp1*	Secreted frizzled-related protein 1	Chr 8	2.19	3.69E-11	1.83	1.18E-05
*Mmp2*	Matrix metallopeptidase 2	Chr 8	2.00	9.80E-15	1.58	2.20E-05
*Ugt2b34*	UDP glucuronosyltransferase 2 family, polypeptide B34	Chr 5	1.90	1.69E-13	1.66	4.46E-07

As shown in Table 2, we acknowledge that the top persistently up-regulated protein-coding DEGs at both days 7 and 14 are nearing baseline levels by day 14. However, among all the 40 persistent DEGs, 5 maintained the same level of fold change or even manifested an increase (Table 3). A gene encoding the solute carrier family 38, member 4 (*Slc38a4*) was among these genes. Rooman *et al*. reported that members of the SLC family (amino acid transporters) may be involved in the injury and recovery phase of the pancreas^43^. Several SLC genes were shown to be expressed in mature acinar cells. The expression of amino acid transporters was proposed to be due to the high demand of digestive enzyme synthesis within mature acinar cells. The up-regulation of the amino acid transporter *Slc38a4* at day 7 and day 14 post-injury coincided with the up-regulation of several trypsinogen digestive enzymes.

**Table 3:** List of the up-regulated protein-coding DEGs between baseline and day 7 whose fold change remained the same or even increased at day 14 (out of 40 DEGs, Fig. 2c).

Gene Symbol	Gene Name	Chromosome	Day 7		Day 14	
			Fold Change	Adjusted *P*-Value	Fold Change	Adjusted *P*-Value
*Ambp*	Alpha 1 microglobulin/bikunin	Chr 4	1.74	3.35E-06	1.89	6.12E-07
*Slc38a4*	Solute carrier family 38, member 4	Chr 15	1.67	5.56E-05	1.69	2.80E-04
*F3*	Coagulation factor III	Chr 3	1.58	8.40E-04	1.64	1.68E-03
*Gc*	Group specific component	Chr 5	1.52	4.42E-03	1.54	1.40E-02
*P2ry1*	Purinergic receptor P2Y, G-protein coupled 1	Chr 3	1.52	2.78E-03	1.58	5.51E-03

### The top down-regulated protein-coding DEGs

Based on the fold change, the top down-regulated protein-coding DEGs between baseline and day 7 tissues and between baseline and day 14 tissues are listed in Table 4. We found syncollin (*Sycn*) to be differentially down-regulated at day 7 post-injury. SYCN is expressed in acinar cells of the exocrine pancreas, where it is bound to the zymogen granule membrane^44^ and regulates exocytosis^45^. *Fgf21* was also differentially down-regulated at day 7 post-injury, and FGF21 has recently been implicated as a secretagogue for digestive enzyme secretion from pancreatic acinar cells^46,47^. While we observed that several trypsinogens were differentially up-regulated at day 7 post-injury, *Prss2* (also known as trypsinogen 20) was differentially down-regulated at day 7 post-injury. The top down-regulated protein-coding DEGs at day 7 that persist at day 14 post-injury are listed in Supplementary Table S5.

**Table 4:** List of the top down-regulated protein-coding DEGs between baseline and day 7 (out of 279 DEGs, Fig. 2c) and between baseline and day 14 (out of 13 DEGs, Fig. 2c).

	Gene Symbol	Gene Name	Chromosome	Fold Change	Adjusted *P*-Value
Day 7	*Shank1*	SH3/ankyrin domain gene 1	Chr 7	0.42	3.75E-27
	*Sycn*	Syncollin	Chr 7	0.49	2.99E-20
	*Hamp2*	Hepcidin antimicrobial peptide 2	Chr 7	0.50	8.79E-13
	*Cyp3a13*	Cytochrome P450, family 3, subfamily a, polypeptide 13	Chr 5	0.54	1.02E-06
	*Fgf21*	Fibroblast growth factor 21	Chr 7	0.54	1.22E-06
	*Reep6*	Receptor accessory protein 6	Chr 10	0.55	6.40E-07
	*Prss2*	Protease, serine 2	Chr 7	0.55	4.47E-18
	*Pnlip*	Pancreatic lipase	Chr 19	0.56	2.28E-29
	*Smim24*	Small integral membrane protein 24	Chr 10	0.56	3.40E-05
	*Cpa1*	Carboxypeptidase A1, pancreatic	Chr 6	0.56	2.41E-22
Day 14	*Nxph2*	Neurexophilin 2	Chr 2	0.64	6.75E-03
	*Foxe3*	Forkhead box E3	Chr 4	0.65	1.61E-02

### DEGs between male and female mice in a histologically recovered pancreas

**Figure 5:**
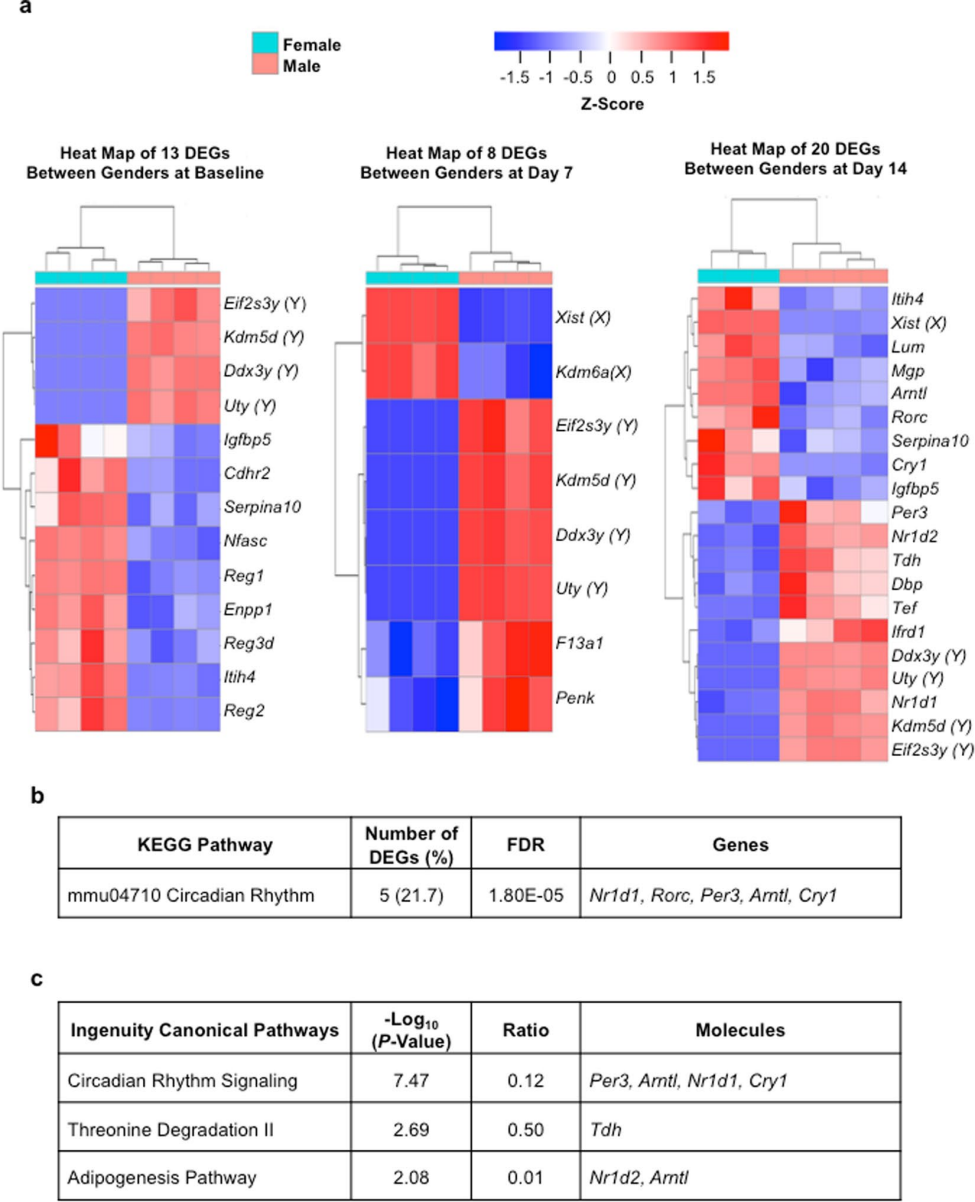
RNA-seq analysis detects DEGs between male and female pancreatic tissue at baseline, day 7, and day 14 post-injury. (**a**) Heat maps illustrate the expression level of the DEGs between male and female mice at baseline, day 7, and day 14 post-injury tissue. The heat map was generated based on the z-score of normalized gene counts from the RNA-seq analysis. Each column is a sample, and each row is a gene. For any given gene, the red color represents gene expression that is greater than the overall mean, and the blue color represents gene expression that is less than the overall mean. Hierarchical clustering of genes and samples are represented by the dendrograms on the left and across the top of the heat map. The statistical criterion for a gene to be considered differentially expressed was a fold-change ≥ 1.5 and an adjusted *p*-value ≤ 0.05. Sex-linked DEGs: Y = Y chromosome; X = X chromosome. (**b**) KEGG pathway analysis and (c) IPA on the non-sex-linked DEGs between male and female mice. The percentage of DEGs in the KEGG pathway analysis is based on the number of non-sex-linked DEGs identified in each pathway out of the total 23 non-sex-linked DEGs. The ratio in the IPA represents the number of non-sex-linked DEGs in each pathway over the total number of genes associated with that pathway.

The overall histological score for inflammatory infiltrate and necrosis did not indicate significant differences in the severity of injury or the subsequent recovery between female and male mice (Supplementary Figure 2). To determine sex differences at the molecular level, the RNA-seq data were analyzed for DEGs between male and female mice at baseline and at days 7 and 14 post-injury. A heat map illustrating the relative expression of 41 DEGs between the sexes and their identity is presented in Fig. 5a. Between male and female mice, several, although not all, of the DEGs can be explained by sex linkage. KEGG pathway analysis and IPA were performed on the 23 non-sex-linked DEGs detected between the sexes to determine possible molecular functions associated with these genes. There were too few genes to perform pathway analysis on each group individually therefore analysis was performed on all 23 non-sex-linked DEGs simultaneously to achieve statistical significance. The enriched pathways and DEGs associated with each pathway are shown in Fig. 5b and c. The circadian rhythm pathway was found to be consistently enriched in each analysis followed by threonine degradation and the adipogenesis pathway. Differential expression of *Itih4* and *Penk* between male and female mice was assessed by RT-qPCR and was found to follow the trend reported in the RNA-seq analysis (Supplementary Figure S3).

## Discussion

The pancreas appears to have a remarkable ability to regenerate and recover after injury^11,12^. In rodents, recovery of the pancreas after mild to moderately severe experimental pancreatitis has been reported to occur within one week after injury^7,10,13–15^. Here, we show that although there is histological resolution of the pancreas one week after injury, there are still numerous DEGs at one or even two weeks after injury. At two weeks post-injury, there was a persistence of differential expression in 12.5% (40 out of 319) of the genes seen one week after injury. Many of the DEGs are associated with replenishing the zymogen granule cargo, tissue remodeling, as well as cellular growth and differentiation suggesting that on a molecular level, the recovery process of the pancreas takes longer than previously appreciated.

It is possible that ultrastructural changes may be present or evolving that cannot be observed by light microscopy on the H&E stained pancreatic tissue. Given that many of the DEGs are associated with replenishing the zymogen granule cargo, tissue remodeling, and cellular growth and differentiation pathways, this might suggest the pancreas may still be recovering at one and even two weeks post injury at both the molecular and ultrastructural level. In addition, it has been reported that islet morphology remains intact^48^ during acute pancreatitis. We too found the morphology of the islets to be structurally intact during injury (Supplementary Figure S4). While light microscopy indicates the islet cells are intact, several of the DEGs in the RNA-seq analysis are associated with the regulation of pancreatic islet cell growth and function, a further indication that the histological resolution of the pancreas by light microscopy does not accurately reflect whether the pancreas has recovered at an ultrastructural or molecular level.

Among the DEGs identified, several were zymogens. The overriding bulk of zymogen genes were trypsinogens, which are the primary digestive proteases^21^. Of the 20 known mouse trypsinogen genes, 11 are thought to encode for functional proteins^49^. However, the level of expression in the mouse pancreas may be strain- and injury-specific^49–51^. While the functional trypsinogens were detected in the baseline pancreas, not all where differentially expressed in the recovering pancreas at the time points sequenced (one and two weeks post-injury). In Tables 1 and 2 we report trypsinogen 4, 5, 12, 15, 16, and mesotrypsin-like among the top differentially up-regulated genes post-injury. It should be noted that trypsinogen 7 and 20 were found to be differentially down-regulated at day 7 but are not shown in Table 4 since they were not amongst the greatest differentially down-regulated DEGs. Whether the differential expression of trypsinogens suggests that the pancreas is still working to replenish its zymogen pool two weeks after injury or whether there is a permanent alteration in trypsinogen expression is not clear. However, if the latter is true, an increase in trypsinogen gene expression in a recovered pancreas could affect severity of future bouts of pancreatitis. Future *in vivo* experiments need to be conducted to further elucidate the role of the differentially expressed trypsinogens during recovery.

We also report the down-regulation of *Sycn* and *Fgf21* one week after injury. The *Sync* product, syncollin, binds to the zymogen granule membrane^44^ and plays a role in regulating granule exocytosis^45^, while FGF21 stimulates enzyme secretion^46,47^. Thus, the process of pancreatic recovery and acinar reconstitution appears to be a highly coordinated event orchestrated by the up-regulation of the zymogen genes themselves and the down-regulation of genes associated with acinar cell exocytosis and secretion. The net effect may be an expedited restoration of the acinar cell zymogen pool.

Of the 332 DEGs, at day 7 there were 8 snoRNAs out of 319 DEGs, and at day 14 there were 12 snoRNAs out of 53 DEGs. These functional non-coding RNAs can be as long as a couple hundred nucleotides, and they are usually transcribed from the intron of a host gene^20,52–55^. After splicing, snoRNAs can escape degradation by forming protein complexes^54^. A primary function identified for snoRNAs is to guide ribosomal RNA modifications for ribosomal biogenesis^20,52,53,55^. The validation of snoRNAs is technically challenging due to limited primer options, secondary structures that may impede the reverse transcription in RT-qPCR, and subcellular localization of snoRNAs. While snoRNAs are primarily localized to the nucleolus, they can also be found in Cajal bodies within the nucleus, or, in response to stress, they can translocate to the cytoplasm^56^. The subcellular localization of snoRNAs may limit the detection of differential expression by RT-qPCR^56^. Despite the snoRNAs not being validated by RT-qPCR, the RNA-seq read coverage, while in itself is not a test of significance, does indicate a change in the snoRNAs gene expression post-injury (Supplementary Figures S5, S6, and S7).

Pancreatic regeneration, whether after injury, partial pancreatectomy, or pregnancy, recapitulates several developmental programs that are observed during embryogenesis of the pancreas. These include, but are not limited to, components of the Notch, Hedgehog, and Wnt signaling pathways as well as the developmental transcription factors PDX1, PTF1A, NGN3, SOX9, and MIST1^57^. Except for *Sfrp1*, a negative regulator of the Wnt signaling pathway, genes associated with the developing pancreas were not found to be differentially expressed at day 7 and day 14 post-injury. A possible explanation for the absence of these factors from the identified DEGs is that these programs are critical post-injury during the regenerative process at a time earlier than the days studied.

To our knowledge, this is the first report on the use of RNA-seq to profile the transcriptome of the recovering pancreas. Others have performed RNA-seq to examine baseline pancreatic tissue or cell types^58,59^. A recent paper by Kong *et al*. described microarray data after caerulein hyperstimulation in mice, and they also examine the pancreas two weeks after injury^60^. Contrary to microarray technology, RNA-seq has a broad dynamic range and greater fidelity for detection of DEGs. However, RNA-seq generates a vast amount of data, which requires extensive computational analysis.

Notably, at two weeks after injury we observed both the persistence and emergence of DEGs. Whether the 53 DEGs detected two weeks after injury are transient or permanent is of great future interest. In addition, pancreatic injury and the recovery process may turn on a novel transcriptomic profile in the pancreas and whether these alterations impose long- or short-term memory of the injurious event is an important question that needs further exploration. A few clinical reports suggest that subsequent bouts of acute pancreatitis are inherently less severe than the initial episode^61–63^. It remains to be determined whether a change in the transcriptional profile post-injury confers some level of protection to the pancreas against future injurious exposures.

In summary, we show that although there is histological resolution by light microscopy one week after injury in mice, there are still numerous DEGs one and even two weeks after injury. The finding of 319 DEGs one week and 53 DEGs two weeks after injury suggests that while light microscopy implies pancreatic recovery, the pancreas is still recovering on a molecular level. There may also be ultrastructural changes that could not be distinguished by light microscopy. Several trypsinogen genes were the most highly up-regulated DEGs at one week post-injury and persistently up-regulated even at two weeks after injury, suggesting that the pancreas is still working to replenish its zymogen pool two weeks after injury and has yet to fully recover. Overall, we found that on a molecular level the actual recovery of the murine pancreas takes longer than initially thought and that light microscopy is not a definitive indicator of pancreatic recovery.

## Methods

### Animals

Caerulein (Sigma-Aldrich) hyperstimulation was induced in both male and female mice of the C57BL/6 strain from Charles River Laboratories, 6- to 8-weeks of age, weighing 20 to 25 g. Caerulein (50 µg/kg) was given by 8 hourly intra-peritoneal injections for two consecutive days. Day 0 was defined as 24 hours after the last injection. Pancreas tissue was collected from the head of the pancreas (less than a 5 mm portion of the pancreas immediately adjacent to the duodenum^64^) at baseline (non-injured state), day 3 (injury), and at days 7 and 14 post-injury. Upon resection, the tissue was immediately partitioned for RNA extraction and tissue fixation. All mice were maintained on a 12-hour light-dark cycle and had free access to standard laboratory chow and water. All methods and animal experiments were performed in accordance with guidelines approved by the University of Pittsburgh School of Medicine and an animal protocol approved by the University of Pittsburgh Institutional Animal Care and Use Committee. All experiments were performed in accordance with relevant guidelines and regulations.

### Pancreatic Histopathology and Image Analysis

Approximately a 3 mm × 3 mm section of tissue from the head of the pancreas was immediately fixed in 4% paraformaldehyde at room temperature for 24 hours. Paraffin-embedded pancreas sections were stained with H&E and graded for severity of injury in a blinded fashion. Ten systematically selected fields at 200× magnification were graded (from 0 to 3) for inflammatory infiltration and necrosis, as described^65,66^.

### RNA extraction

Total RNA was immediately extracted from 60 mg of tissue from the head of the pancreas using TRIzol (Ambion).  Sixty mg of pancreas tissue yielded 200–300 µg of total RNA. RNA was treated with DNase I (Zymo Research) at room temperature for 20 minutes. The concentration of DNase I-treated RNA was measured using a Qubit 3.0 Fluorometer (Thermo Fisher Scientific), and the integrity of the DNase I-treated RNA was analyzed on the Agilent TapeStation. All DNase I-treated RNA samples used for RNA library construction had RNA Integrity Numbers (RINs) above 6.4 (Supplementary Table S6).

### RNA library construction and next-generation sequencing

RNA libraries were prepared from pancreas samples derived from three groups of mice consisting of both male and female mice: (1) baseline (non-injured), n = 8; (2) day 7, n = 8; and (3) day 14, n = 7. One female mouse in the day 14 group was omitted from all RNA-seq analysis due to poor mapping rates (Supplementary Table S7). For this reason, the day 14 group had 7 mice (3 female and 4 male) while the day 7 and baseline group had 8 mice each (4 male and 4 female). To minimize the effect of any RNA degradation present in the samples, libraries for RNA-seq were generated from ribosomal RNA depleted total RNA rather than from mRNA isolated by poly-A selection. One microgram of each DNase I-treated RNA sample was used for library construction using the Illumina TruSeq Stranded Total RNA Library Prep Kit with Ribo-Zero Gold per the manufacturer’s protocol. Adaptor-ligated fragments were amplified by PCR for 9 cycles. The quality and size of the final library preparations were analyzed on an Agilent TapeStation. Sequencing was performed on the Illumina NextSeq. 500 NGS platform, generating approximately 40 million paired-end 75 bp reads for each sample.

### Preprocessing of the RNA-seq data and differential expression (DE) analysis

Quality control for raw fastq files were performed with FastQC^67^, the low quality reads and 3′ adapters were trimmed with Trim Galore! and Cutadapt^68,69^. Then the trimmed reads were aligned to reference mouse genome (mm10) with the RNA-seq aligner STAR^70^. Subsequently, gene expression for each sample was quantified by counting the number of read fragments that were uniquely mapped to genes using the featureCounts^71^. DEGs were analyzed based on the raw count table using DESeq. 2^72^. When comparing between different time points, gender was adjusted as a covariate; while comparing between genders, the recovery time was adjusted as a covariate. RNA-seq mapping statistics are provided in Supplementary Table S7.

The alignment results (bam files) were converted to bigWig format using the *bam2wig* tool of RSeQC^73^, for the visualization of read coverage in Integrated Genomics Viewer (IGV) genome browser^74^. The total read coverage of each sample was normalized to 10Mbp, so that the peaks of different samples in the IGV plots are comparable.

### Pathway enrichment analysis

The enrichment of DEGs in KEGG pathways and GO terms were analyzed using the Fisher’s exact test. Ingenuity Pathway Analysis (IPA) was also performed on the DEGs to identify enriched canonical pathways and gene networks^75^.

### Real-time quantitative PCR (RT-qPCR)

Real-time quantitative PCR (RT-qPCR) was performed on RNA extracted from pancreas tissue of mice at baseline (non-injured) and at days 7 and 14 post-injury. DNase I-treated RNA was converted to cDNA using the iScript Reverse Transcription Supermix for RT-qPCR (Bio-Rad) per the manufacturer’s instruction. qPCR was performed with SsoAdvanced Universal SYBR Green Supermix (Bio-Rad) on a Bio-Rad CFX Connect Real-Time System at an annealing temperature of 55 °C. Intron spanning gene-specific primers were purchased from IDT utilizing the IDT PrimeTime Predesigned qPCR assays tool: *Gm2663* forward 5′-GACATGTGTAGCCTCCAACA-3′, *Gm2663* reverse 5′-CAGACACACTGAAAGACTATGAAGA-3′; *Agr2* forward 5′-GTCAGAAGGTTCATAAGCGTAGA-3′, *Agr2* reverse 5′-GACAAGCACCTTTCTCCTGAT-3′; *Snora16a* forward 5′-GTGGCCCTTATCGAAGCTG-3′, *Snora16a* reverse 5′-GAAACAGAACTGCGACCGT-3′; *Snora41* forward 5′-AGCTGTCTTTATGGTAGCAGT-3′, *Snora41* reverse 5′-GTGTTTCTAGGATGCTCTGGT-3′; *Snora52* forward 5′-GGTCCATCCTAATACCTGCTG-3′, *Snora52* reverse 5′-GTGTTTCTAGGATGCTCTGGT-3′; Rplp0 forward 5′-TTATAACCTGAAGTGCTCGAC-3′, *Rplp0* reverse 5′-CGCTTGTACCCATTGATGATG-3′. Primers were also purchased from Bio-Rad utilizing the PrimePCR tool: *Sycn* (Assay ID: qMmuCED0003504), *Itih4* (Assay ID: qMmuCID0023139), and *Penk* (Assay ID: qMmuCED0050607). PCR efficiency for all primers was 95% or greater, and specificity was confirmed by a single peak in melt curve analysis. The relative gene expression for each gene of interest was determined using the 2-ΔΔCt method. All values were normalized to *Rplp0*, a control gene that was confirmed to have consistent gene expression across sample groups (Supplementary Table S8).

### Data availability

The datasets generated during and analyzed during the current study are available in the GEO repository.

https://www.ncbi.nlm.nih.gov/geo/query/acc.cgi?acc=GSE99774

### Accession number

GSE99774.

The following secure token has been created to allow review of record GSE99774 while it remains in private status: kzwjgoiarboddeb.

## Electronic supplementary material

Supplemental Materials
