# Supplementary material for: Pancreatic gene expression during recovery after pancreatitis reveals unique transcriptome profiles
Source: Sci Rep. 2018 Jan 23;8:1406. doi: 10.1038/s41598-018-19392-0 (PMC5780441; doi:10.1038/s41598-018-19392-0)

Pancreatic gene expression during recovery after pancreatitis reveals unique transcriptome profiles

Kristy Boggs[+1], Ting Wang[+1,3], Abrahim I. Orabi[1], Amitava Mukherjee[1], John F. Eisses[1], Tao Sun[3], Li Wen[1], Tanveer A. Javed[1], Farzad Esni[2], Wei Chen[1,3], Sohail Z. Husain*[1]

Departments of [1]Pediatrics and [2]Surgery, School of Medicine and [3]Department of Biostatistics, School of Public Health, University of Pittsburgh, Pittsburgh, PA, 15224 USA

Correspondence and requests for materials should be addressed to Sohail Z. Husain (sohail.husain@chp.edu)

SUPPLEMENTARY FIGURE LEGENDS

Supplementary Figure S1. Experimental validation of DEGs between male and female mice from RNA-seq analysis by RT-qPCR. (a, d) Relative expression of *Snora16* by RT-qPCR and as FPKM values by RNA-seq. (b, e) Relative expression of *Snora41* by RT-qPCR and FPKM values by RNA-seq. (c, f) Relative expression of *Snora52* by RT-qPCR and FPKM values by RNA-seq. Error bars represent ± s.d.

Supplementary Figure S2. The overall histologic severity scoring of H&E-stained paraffin sections from the head of the pancreas for inflammation and necrosis between the sexes at baseline, day 7, and day 14 post-injury. Error bars represent ± s.d.

Supplementary Figure S3. Experimental validation of DEGs between male and female mice from RNA-seq analysis by RT-qPCR. (a, c) Relative expression of *Penk* by RT-qPCR and as FPKM values by RNA-seq. (b, d) Relative expression of *Itih4* by RT-qPCR and FPKM values by RNA-seq. Overall, the trend of gene expression by RT-qPCR mirrors that of the RNA-seq analysis. Error bars represent ± s.d.

Supplementary Figure S4. The islet of Langerhans in baseline and day 3 (injury) H&E-stained paraffin sections of the pancreas. The black arrows point to the islets.

Supplementary Figure S5. Integrated Genomics Viewer plot showing RNA-seq read coverage for *Snora16a.* The genomic location is shown across the top. The middle section shows read coverage for the baseline (track 1-8, gray color), day 7 (track 9-16, red color), and day 14 (track 17-23, blue color). The gene structure is shown at the bottom.

Supplementary Figure S6. Integrated Genomics Viewer plot showing RNA-seq read coverage for *Snora41.* The genomic location is shown across the top. The middle section shows read coverage for the baseline (track 1-8, gray color), day 7 (track 9-16, red color), and day 14 (track 17-23, blue color). The gene structure is shown at the bottom.

Supplementary Figure S7. Integrated Genomics Viewer plot showing RNA-seq read coverage for *Snora52.* The genomic location is shown across the top. The middle section shows read coverage for the baseline (track 1-8, gray color), day 7 (track 9-16, red color), and day 14 (track 17-23, blue color). The gene structure is shown at the bottom.

Supplementary Table S1

	Gene Symbol	Gene Name	Chromosome	Fold Change	Adjusted *P*-Value
**Day 7**	*Snora23a*	Small nucleolar RNA, H/ACA box 23	Chr 6	1.58	8.39E-10
	Snora64	Small nucleolar RNA, H/ACA box 64	Chr 6	1.55	1.88E-05
	Sora31	Small nucleolar RNA, H/ACA box 31	Chr 6	1.52	3.00E-04
**Day 14**	*Snord49a*	Small nucleolar RNA, C/D box 49A	Chr 11	1.90	3.43E-14
	Snora16a	Small nucleolar RNA, H/ACA box 16A	Chr 4	1.63	3.94E-06
	Snora52	Small nucleolar RNA, H/ACA box 52	Chr 7	1.58	5.87E-04
	Snord88a	Small nucleolar RNA, C/D box 88A	Chr 7	1.55	9.05E-03
	Snord49b	Small nucleolar RNA, C/D box 49B	Chr 11	1.53	2.82E-04
	Snora33	Small nucleolar RNA, H/ACA box 33	Chr 10	1.51	7.06E-05
	Snora17	Small nucleolar RNA, H/ACA box 17	Chr 2	1.51	2.20E-05

Supplementary Table S1. List of the top up-regulated non-coding RNA DEGs between baseline and day 7 (out of 279 DEGs, Fig. 2c) and between baseline and day 14 (out of 13 DEGs, Fig. 2c).

Supplementary Table S2

Gene Symbol	Gene Name	Chromosome	Day 7			Day 14	
			Fold Change	Adjusted *P*-Value		Fold Change	Adjusted *P*-Value
Snora26	Small nucleolar RNA, H/ACA box 26	Chr 5	1.91	2.62E-15		1.74	1.61E-09
Snora41	Small nucleolar RNA, H/ACA box 41	Chr 1	1.86	1.03E-12		1.93	2.28E-12
Snora68	Small nucleolar RNA, H/ACA box 68	Chr 8	1.68	1.41E-09		1.51	4.19E-05
Snord65	Small nucleolar RNA, C/D box 65	Chr 11	1.56	3.87E-04		1.62	5.87E-04
Snord35a	Small nucleolar RNA, C/D box 35A	Chr 7	1.56	7.03E-04		1.65	6.75E-04

Supplementary Table S2. List of the top up-regulated non-coding RNA DEGs at day 7 that remain up-regulated at day 14 (out of 40 DEGs, Fig. 2c).

Supplementary Table S3

	KEGG Pathway	Number of DEGs (%)	FDR	Genes
Day 7	mmu04974 Protein digestion and absorption	17 (5.3)	1.29E-10	*1810009J06Rik,Gm10334,Cpa1,Prss2,Col5a2,Col6a1, 2210010C04Rik,Col6a3,Cela3b,Ctrb1,Ctrl,Col3a1, Col14a1,Col1a2,Col5a1,Slc16a10,Eln*
	mmu05144 Malaria	8 (2.5)	2.00E-04	*Hba-a1,Hba-a2,Hbb-bt,Hbb-b2,Hgf,Hbb-bs,Hbb-b1,Tlr4*
	mmu04610 Complement and coagulation cascades	10 (3.1)	2.00E-04	*Plau,Pros1,C1qa,C3ar1,C1s1,Serping1,C1ra,F3,C1qc,C1qb*
	mmu04972 Pancreatic secretion	10 (3.1)	3.00E-04	*1810009J06Rik,Gm10334,Pnlip,Cpa1,Prss2, 2210010C04Rik,Cela3b,Ctrb1,Ctrl,Car2*
	mmu04151 PI3K-Akt signaling pathway	17 (5.3)	8.00E-04	*Lama2,Col6a1,Pck2,Pdgfra,Col6a3,Flt1,Col1a2,Spp1, Fgf21, G6pc2,Gng7,Pdgfc,Ins1,Hgf,Ins2,Tlr4,Fgf12*
	mmu05150 Staphylococcus aureus infection	7 (2.2)	8.00E-04	*C1qa,C3ar1,C1s1,Fcgr3,C1ra,C1qc,C1qb*
	mmu05143 African trypanosomiasis	6 (1.9)	9.90E-04	*Hba-a1,Hba-a2,Hbb-bt,Hbb-b2,Hbb-bs,Hbb-b1*
	mmu04950 Maturity onset diabetes of the young	5 (1.6)	2.24E-03	*Slc2a2,Iapp,Ins1,Ins2,Neurod1*
	mmu05133 Pertussis	7 (2.2)	4.07E-03	*C1qa,C1s1,Serping1,C1ra,C1qc,Tlr4,C1qb*
	mmu04014 Ras signaling pathway	11 (3.4)	1.55E-02	*Pdgfra,Flt1,Fgf21,Pla1a,Pla2g4a,Gng7,Pdgfc,Ins1,Hgf, Ins2,Fgf12*
	mmu04512 ECM-receptor interaction	6 (1.9)	3.67E-02	*Lama2,Col6a1,Col6a3,Col1a2,Spp1,Cd44*
Day 14	mmu04918 Thyroid hormone synthesis	2 (3.8)	3.94E-02	*Alb,Iyd*
	mmu00830 Retinol metabolism	2 (3.8)	3.94E-02	*Ugt2b34,Dhrs9*
	mmu05205 Proteoglycans in cancer	3 (5.7)	3.94E-02	*Mmp2,Lum,Plau*
	mmu04080 Neuroactive ligand-receptor interaction	3 (5.7)	3.94E-02	*1810009J06Rik,Gm10334,P2ry1*
	mmu04974 Protein digestion and absorption	2 (3.8)	3.94E-02	*1810009J06Rik,Gm10334*
	mmu04610 Complement and coagulation cascades	2 (3.8)	3.94E-02	*Plau,F3*
	mmu04972 Pancreatic secretion	2 (3.8)	3.97E-02	*1810009J06Rik,Gm10334*

Supplementary Table S3. Enrichment of KEGG pathways for the DEGs. The number and percentage of DEGs in each significant pathway (FDR ≤ 0.05), as well as the identity of the DEGs, are listed in the table. The percentage of DEGs is based on the number of DEGs identified in each pathway out of the total number of DEGs between baseline and day 7 (319 DEGs) and baseline and day 14 (53 DEGs).

Supplementary Table S4

	Ingenuity Canonical Pathways	$-Log_{10}$ (*P*-Value)	Ratio	Molecules
Day 7	Hepatic Fibrosis / Hepatic Stellate Cell Activation	7.07	0.09	*Col5a2,Flt1,Mmp2,Il1r1,Pdgfc,Col1a2,Col5a1,Tlr4,Col6a1,Col6a3, Timp1,Hgf,Pdgfra,Lbp,Col3a1*
	Complement System	6.27	0.21	*C1r,Serping1,C1s,C1qc,C1qa,C1qb,C3ar1*
	Atherosclerosis Signaling	4.20	0.08	*Col1a2,Pla2g4a,Apoe,Lyz,Ccr2,Pla2g7,F3,Pdgfc,Col3a1*
	Inhibition of Matrix Metalloproteases	3.63	0.14	*Adam12,Timp1,Mmp14,Mmp2,Mmp19*
	Maturity Onset Diabetes of Young (MODY) Signaling	3.59	0.19	*Neurod1,Slc2a2,Ins,Ins1*
	LXR/RXR Activation	3.55	0.07	*Tlr4,Apoe,Lyz,Ambp,Serpinf1,Il1r1,Lbp,Gc*
	PXR/RXR Activation	3.52	0.10	*Cyp3a7,Pck2,Ins,Hmgcs2,Ins1,Abcb9*
	FXR/RXR Activation	3.42	0.07	*G6pc2,Apoe,Pck2,Ambp,Ins,Serpinf1,Gc,Ins1*
	Bladder Cancer Signaling	2.72	0.07	*Fgf21,Mmp14,Fgf12,Mmp2,Pdgfc,Mmp19*
	Eicosanoid Signaling	2.54	0.08	*Pla2g4a,Alox5ap,Hpgds,Tbxas1,Pla2g7*
	NF-κB Signaling	2.30	0.05	*Tlr4,Ntrk2,Flt1,Ins,Pdgfra,Fcer1g,Il1r1,Ins1*
	HIF1α Signaling	2.13	0.05	*Slc2a5,Mmp14,Slc2a2,Mmp2,Pdgfc,Mmp19*
	Prostanoid Biosynthesis	2.11	0.22	*Hpgds,Tbxas1*
	Intrinsic Prothrombin Activation Pathway	2.11	0.11	*Col1a2,Pros1,Col3a1*
Day 14	LXR/RXR Activation	2.92	0.03	*Alb,Ambp,Gc*
	FXR/RXR Activation	2.87	0.03	*Alb,Ambp,Gc*
	Coagulation System	2.69	0.06	*Plau,F3*
	Serotonin Degradation	2.26	0.03	*Dhrs9,Ugt2b10*
	Thyroid Hormone Biosynthesis	2.24	0.33	*Iyd*
	Glioma Invasiveness Signaling	2.12	0.03	*Mmp2,Plau*

Supplementary Table S4. Ingenuity Pathway Analysis (IPA) on the DEGs detects significantly over-represented canonical pathways. The set of DEGs associated with each pathway have a p-value ≤ 0.05. The ratio represents the number of DEGs in each pathway over the total number of genes associated with that pathway.

Supplementary Table S5

Gene Symbol	Gene Name	Chromosome	Day 7			Day 14	
			Fold Change	Adjusted *P*-Value		Fold Change	Adjusted *P*-Value
Lars2	Leucyl-tRNA synthetase, mitochondrial	Chr 9	0.49	1.10E-15		0.61	1.31E-06
Nsg1	Neuron specific gene family member 1	Chr 5	0.51	1.70E-09		0.63	5.36E-04
Iyd	Iodotyrosine deiodinase	Chr 10	0.56	4.93E-07		0.60	1.61E-04
Fxyd6	FXYD domain-containing ion transport regulator 6	Chr 9	0.60	1.25E-09		0.66	1.16E-05
Cux2	Cut-like homeobox 2	Chr 5	0.64	8.08E-05		0.66	2.43E-03

Supplementary Table S5. List of the top down-regulated protein-coding DEGs at day 7 that remain down-regulated at day 14 (out of 40 DEGs, Fig. 2c).

Supplementary Table S6

Group	RNA Integrity Number (RIN)							
**Baseline**	7.1	6.9	7.0	6.9	7.1	6.9	7.5	7.5
**Day 7**	7.3	6.6	7.8	7.4	7.7	7.1	6.7	7.7
**Day 14**	6.6	6.9	6.4	7.3	6.5	6.6	6.9	6.8

QC Parameters	Correlation with RIN Values	*P*-Value
Mapping Rate (%)	-0.155	0.481
Duplicate Rate (%)	0.239	0.272
Exon Tags (%)	-0.105	0.632
Intron Tags (%)	0.019	0.933
# of Genes (count>0)	-0.338	0.114
GE.PC1	-0.346	0.106
GE.PC2	-0.348	0.103
GE.PC3	-0.199	0.363
GE.PC4	-0.118	0.592
GE.PC5	0.254	0.242
GE.PC6	0.329	0.125
GE.PC7	-0.235	0.281
GE.PC8	-0.18	0.411
GE.PC9	0.059	0.79
GE.PC10	-0.393	0.063

Supplementary Table S6. (Top) RNA Integrity Number (RIN) used to assess the quality of RNA extractions from pancreas tissue. The pancreas is one of the most difficult tissues from which to extract quality RNA. Despite this difficulty, the RNA samples had RIN values ranging from 6.4 – 7.8. (Bottom) There was no significant correlation found between the RNA RIN values and the mapping characteristics or principal components (PC) of normalized gene counts. This indicates that the integrity of the RNA did not significantly affect mapping or the overall data patterns within the dataset. A *p*-value ≤ 0.05 is considered significant.

Supplementary Table S7

Sample	Group	Sex	Total Reads	Trimmed Reads	Mapping Rate	Duplicate Rate	CDS Exon Tags	5'UTR Exon Tags	3'UTR Exon Tags	Intron Tags	TSS Up 10kb	TES Down 10kb	Gene Count
1	Baseline	F	35188968	388261	79.13%	41.79%	66.36%	3.99%	5.45%	14.68%	8.74%	0.78%	14988
2	Baseline	F	36279847	377861	78.41%	41.48%	65.92%	4.17%	5.53%	14.70%	8.85%	0.83%	15243
3	Baseline	F	35861188	309039	78.69%	40.43%	66.51%	3.93%	5.51%	14.17%	9.06%	0.82%	15348
4	Baseline	F	37175439	428760	78.09%	41.40%	64.53%	4.65%	5.95%	15.90%	8.01%	0.96%	15645
5	Baseline	M	36374162	375190	83.41%	41.87%	65.62%	4.68%	5.66%	13.93%	9.19%	0.92%	15947
6	Baseline	M	33713804	435822	78.53%	41.62%	67.86%	4.19%	5.66%	12.91%	8.59%	0.79%	14780
7	Baseline	M	34900067	492625	77.98%	42.09%	66.72%	4.62%	5.86%	13.66%	8.27%	0.86%	14894
8	Baseline	M	31046796	373170	78.47%	42.22%	65.91%	4.54%	5.82%	14.73%	8.11%	0.89%	14656
9	Day 7	F	36395276	324273	84.88%	37.91%	66.33%	4.24%	6.76%	12.96%	8.63%	1.07%	15813
10	Day 7	F	39099425	311612	85.17%	34.86%	64.25%	4.00%	7.02%	14.66%	8.92%	1.16%	16733
11	Day 7	F	37558397	388059	81.46%	37.57%	66.86%	3.77%	6.11%	13.16%	9.17%	0.92%	15749
12	Day 7	F	34256664	307830	83.53%	35.88%	65.82%	4.02%	6.69%	13.71%	8.71%	1.04%	15842
13	Day 7	M	38100073	347868	83.99%	37.42%	66.90%	4.24%	6.63%	12.50%	8.68%	1.04%	15885
14	Day 7	M	36937129	537934	82.91%	37.39%	64.08%	4.64%	7.30%	14.40%	8.47%	1.12%	16135
15	Day 7	M	33785808	430409	85.79%	34.54%	67.65%	3.66%	6.81%	11.76%	9.08%	1.03%	16439
16	Day 7	M	34510922	541075	83.52%	36.28%	67.79%	3.55%	6.49%	12.04%	9.19%	0.94%	15638
17	Day 14	F	37377223	514541	84.44%	35.29%	70.49%	2.25%	5.72%	11.81%	8.97%	0.75%	15688
18	Day 14	F	38771737	476627	83.73%	35.83%	70.72%	2.43%	5.70%	11.84%	8.56%	0.75%	15704
19	Day 14	F	38634158	462388	82.88%	35.66%	69.81%	2.36%	5.66%	12.78%	8.65%	0.74%	16311
20	Day 14	F	34853096	470625	51.00%	48.65%	60.32%	3.31%	3.96%	25.13%	6.64%	0.64%	14995
21	Day 14	M	37590562	489137	82.34%	36.52%	68.49%	2.44%	5.73%	14.01%	8.48%	0.84%	16342
22	Day 14	M	37915996	554098	81.46%	39.06%	69.08%	2.75%	5.81%	13.68%	7.89%	0.80%	15365
23	Day 14	M	38277351	525804	80.66%	40.87%	68.79%	2.81%	5.73%	13.26%	8.65%	0.77%	15260
24	Day 14	M	38576559	468278	84.88%	36.95%	71.68%	2.20%	5.91%	10.95%	8.49%	0.77%	15551

Supplementary Table S7. Mapping statistics for all RNA-seq samples. Highlighted in grey is a female sample from the day 14 group that was excluded from all RNA-seq data analysis due to poor mapping rates. F, female; M, male.

Supplementary Table S8

Animal Group	Sex	Control Gene	Average Ct Values
Baseline	Female	*Rplp0*	22.70
Baseline	Female	*Rplp0*	22.99
Baseline	Female	*Rplp0*	22.54
Baseline	Female	*Rplp0*	24.61
Baseline	Male	*Rplp0*	23.30
Baseline	Male	*Rplp0*	23.13
Baseline	Male	*Rplp0*	24.00
Baseline	Male	*Rplp0*	23.78
Day 7	Female	*Rplp0*	22.24
Day 7	Female	*Rplp0*	22.12
Day 7	Female	*Rplp0*	22.18
Day 7	Female	*Rplp0*	22.26
Day 7	Male	*Rplp0*	21.28
Day 7	Male	*Rplp0*	20.44
Day 7	Male	*Rplp0*	19.64
Day 7	Male	*Rplp0*	21.81
Day 14	Female	*Rplp0*	23.83
Day 14	Female	*Rplp0*	22.03
Day 14	Female	*Rplp0*	24.29
Day 14	Female	*Rplp0*	21.51
Day 14	Male	*Rplp0*	22.90
Day 14	Male	*Rplp0*	24.26
Day 14	Male	*Rplp0*	22.63
Day 14	Male	*Rplp0*	22.76

Supplementary Table S8. Ct values for the control gene (*Rplp0*) at baseline, day 7, and day 14 post-injury.

Supplementary Figure S1

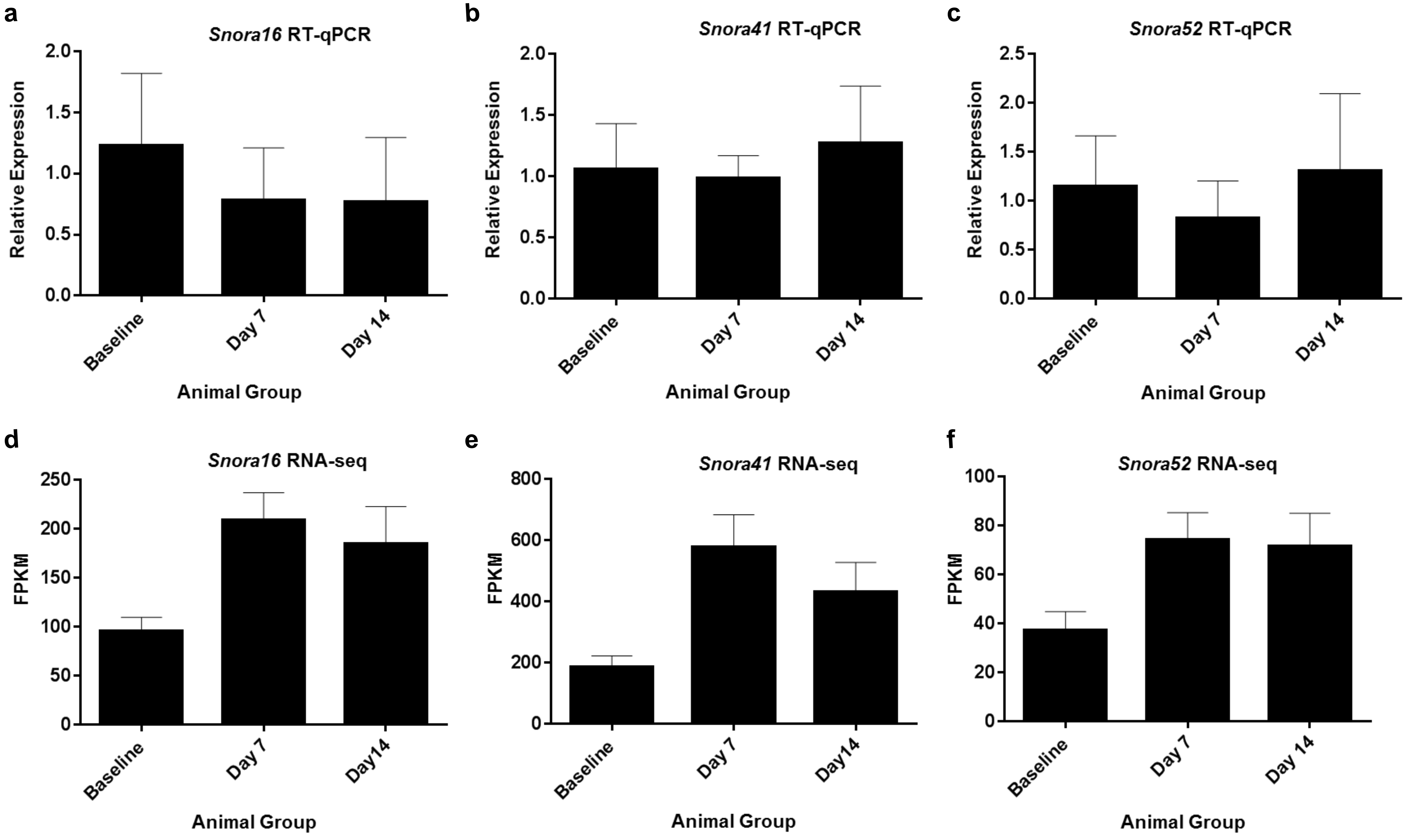

Supplementary Figure S2

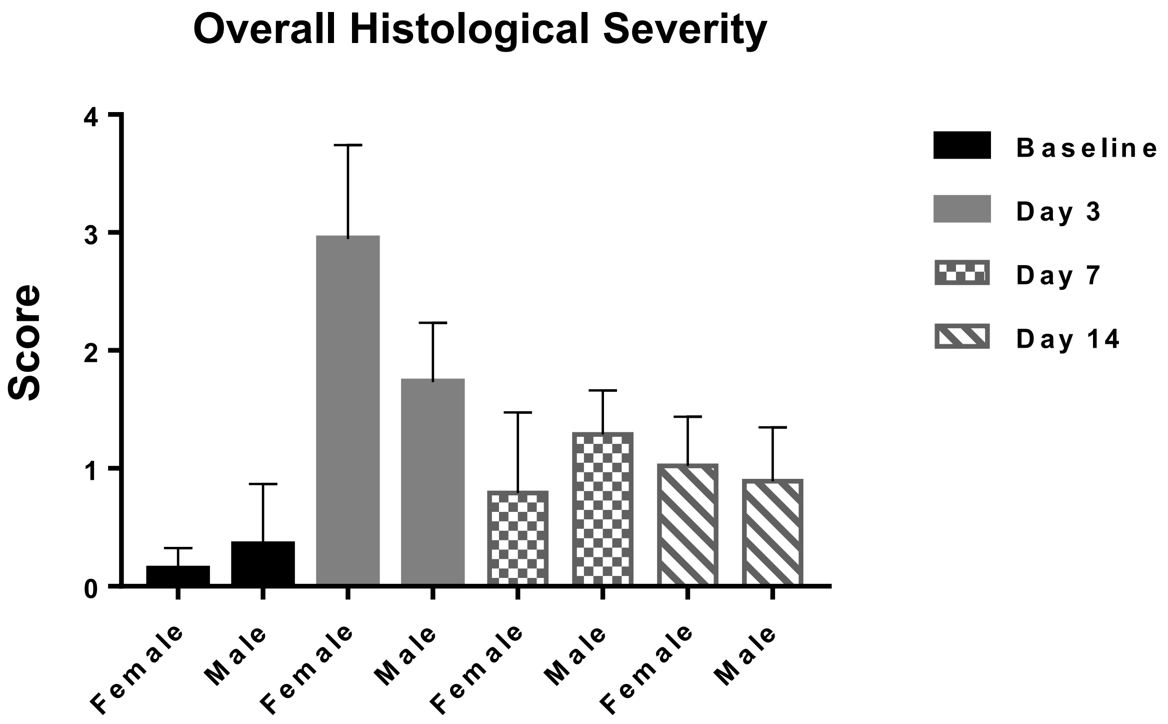

Supplementary Figure S3

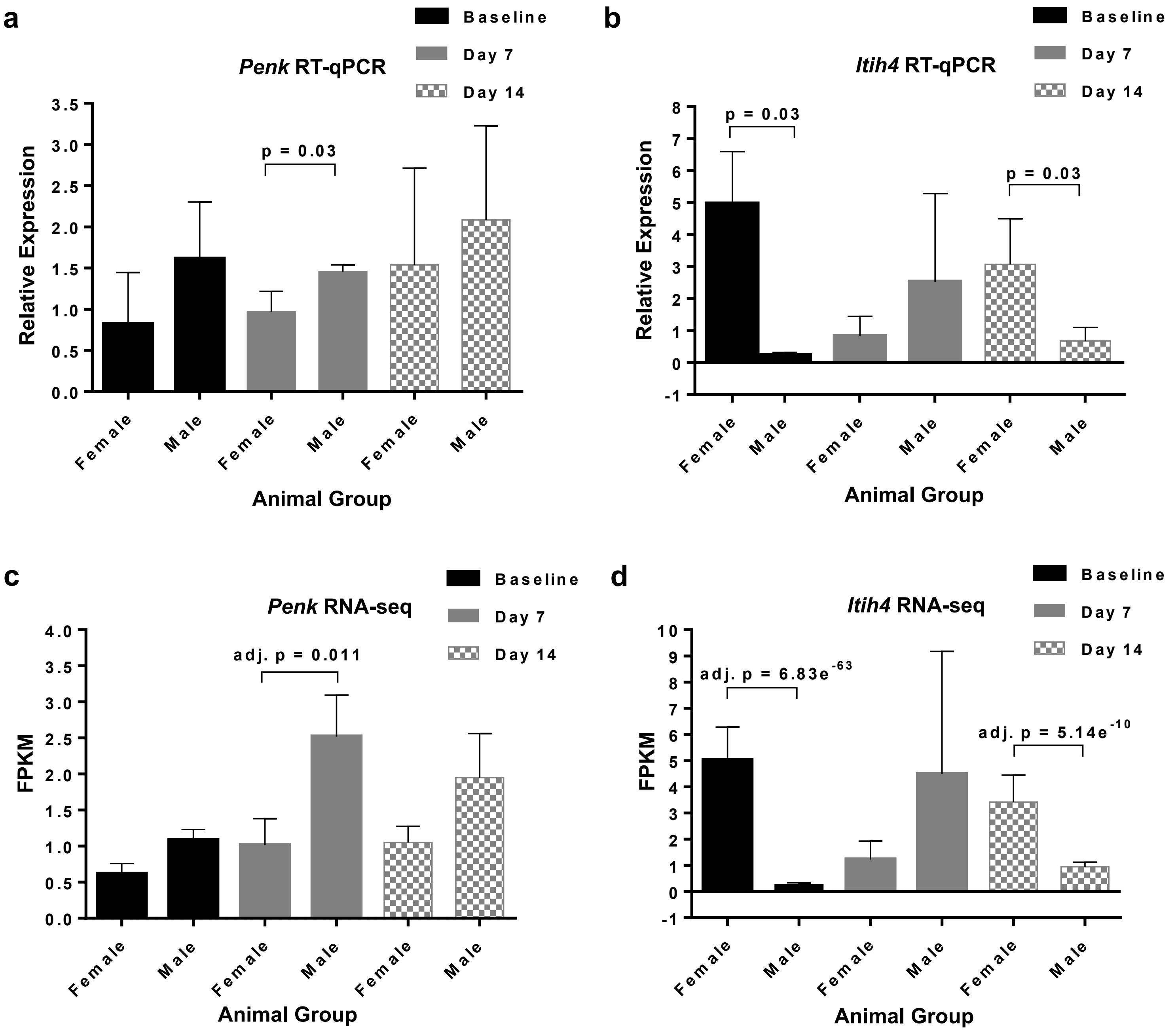

Supplementary Figure S4

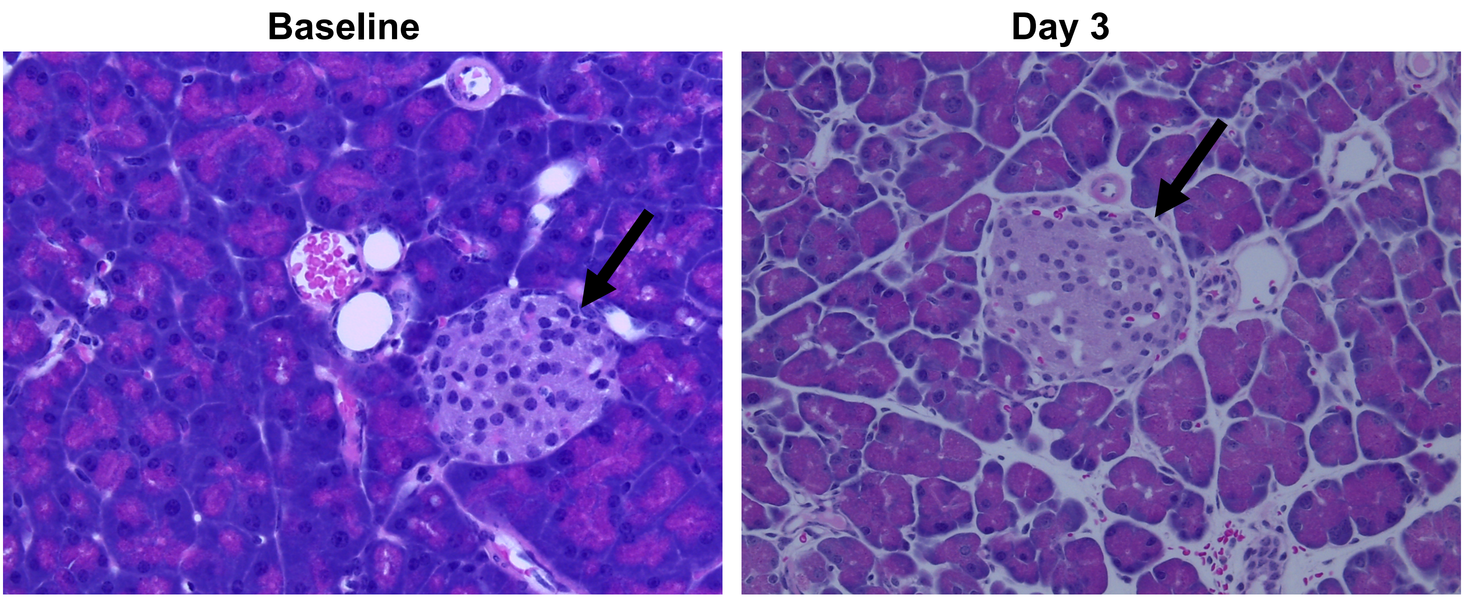

Supplementary Figure S5

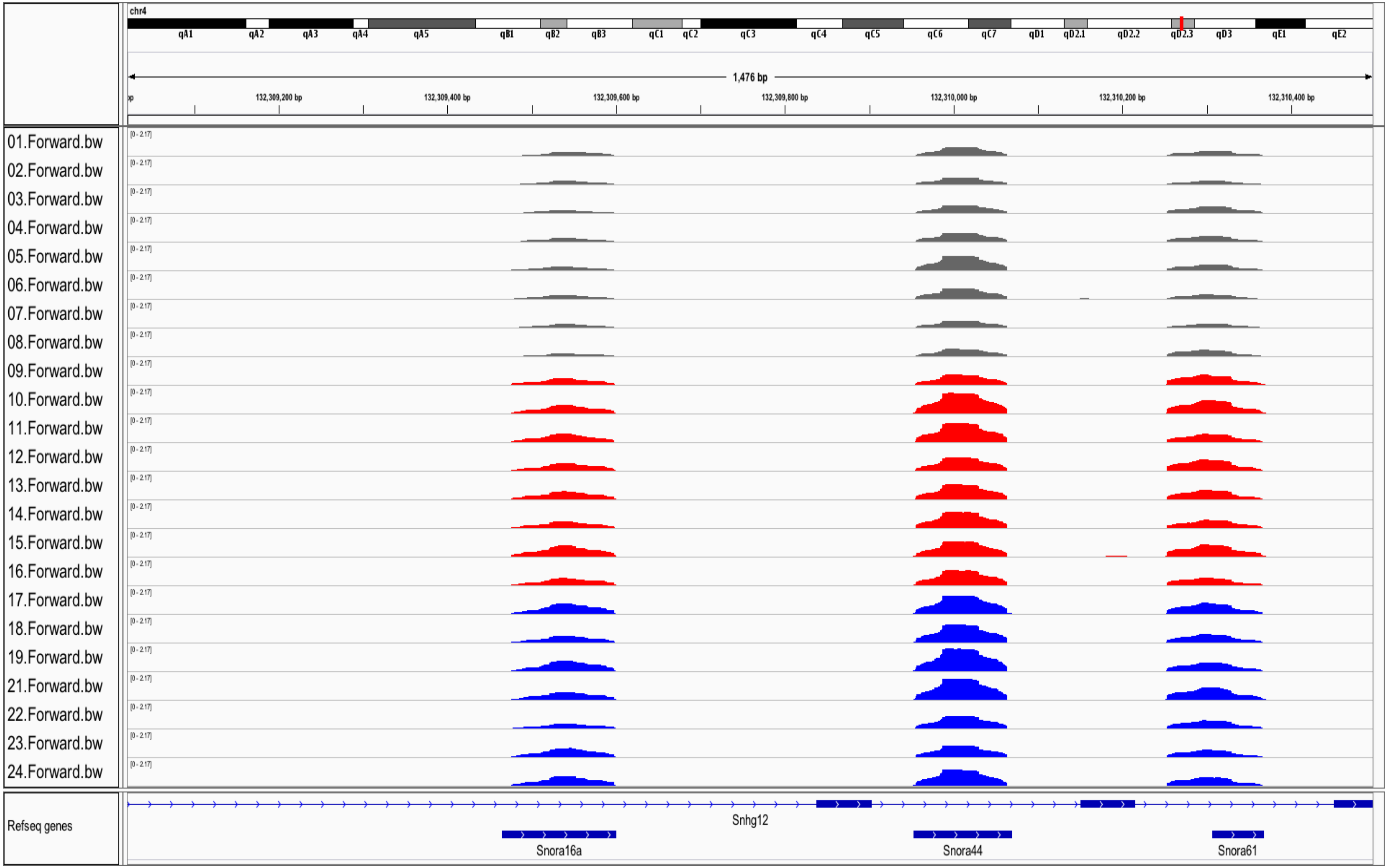

Supplementary Figure S6

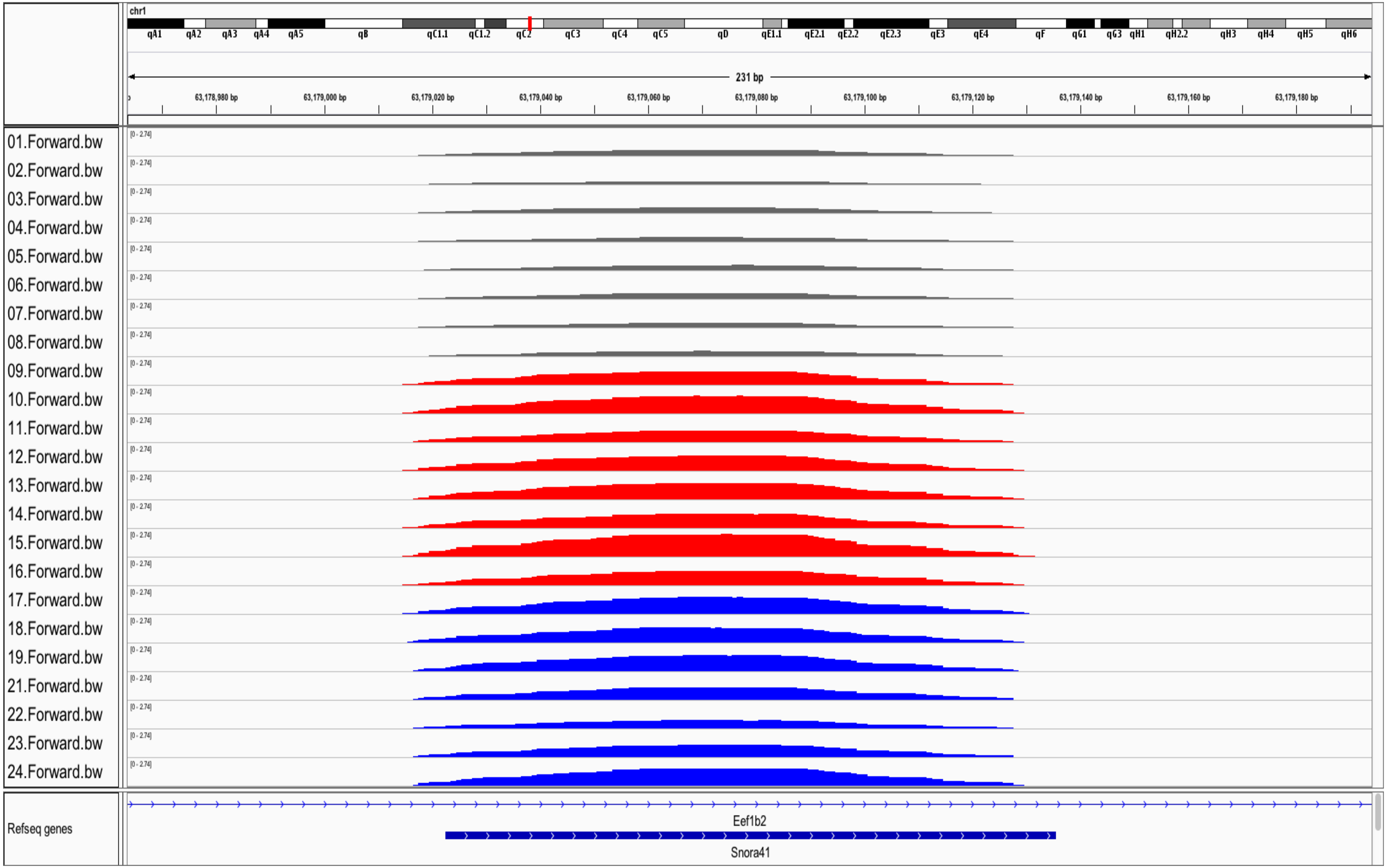

Supplementary Figure S7

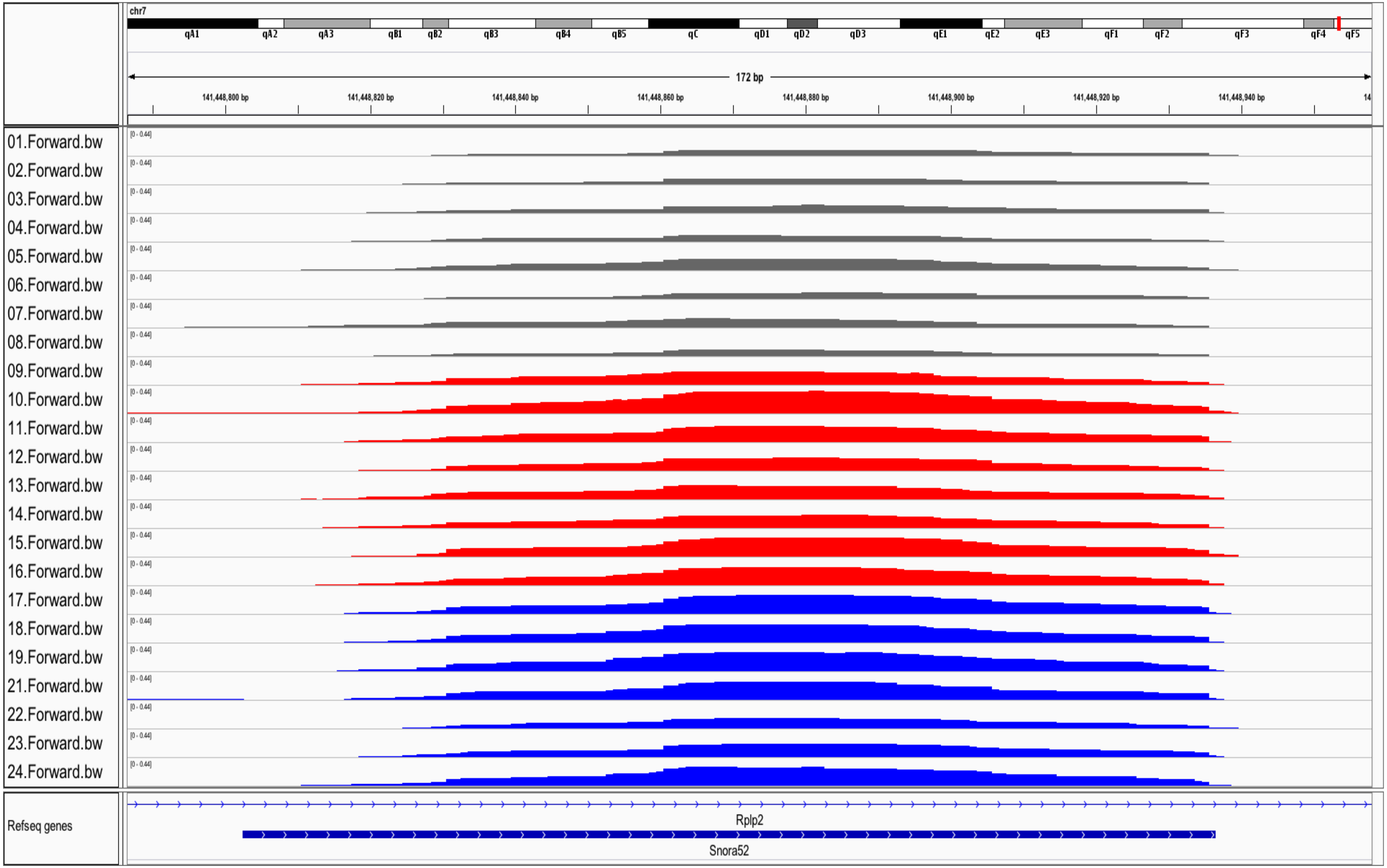

Supplement: Supplementary file 1 — Supplemental Materials [file 41598_2018_19392_MOESM1_ESM.pdf]
